# Chinese massage therapy (Tuina) inhibits motor neuron apoptosis in rats with sciatic nerve injury by regulating the cPLA2 and RhoA/ROCK2 signaling pathways

**DOI:** 10.3389/fneur.2025.1622602

**Published:** 2025-07-30

**Authors:** Jiawei Sun, Yingqi Zhang, Zhifeng Liu, Hanyu Zhang, Jiayue Liu, Yue Xu, Rentuya Na, Hongzheng Zhang, Jiawang Yan, Tianyuan Yu

**Affiliations:** ^1^School of Acupuncture-Moxibustion and Tuina, Beijing University of Chinese Medicine, Beijing, China; ^2^Department of Tuina and Pain Management, Dongzhimen Hospital, Beijing University of Chinese Medicine, Beijing, China

**Keywords:** peripheral nerve injury, Tuina, apoptosis, inflammatory response, CPLA2, RhoA/ROCK2 pathway

## Abstract

**Objective:**

To investigate whether Tuina therapy alleviated inflammation and motor neuron apoptosis in sciatic nerve injury (SNI) rats by regulating cytosolic phospholipase A2 (cPLA2) and Ras homolog family member A/Rho-associated coiled-coil comprising protein kinase 2 (RhoA/ROCK2) signaling cascades.

**Methods:**

Four experimental cohorts were established utilizing 36 male Sprague–Dawley rats: control, sham, SNI, and TUI. We implemented a sciatic nerve injury (SNI) model. At dthe mid-thigh level, sciatic nerves were exposed and crushed for 5 s using non-serrated forceps at points spaced approximately 2 mm apart. Postoperatively, Tuina therapy (Chinese therapeutic massage, Tuina) was administered to evaluate its neuromodulatory effects. SNI models were established in the SNI and TUI cohorts. TUI cohorts applied with “Three-Manipulation and Three-Acupoint” technique, which included pressing, plucking, and kneading on the acupoints Yinmen (BL37), Chengshan (BL57), and Yanglingquan (GB34). The control cohort underwent no intervention. The sham surgery and model cohorts underwent restraining interventions. Motor function was assessed using Basso, Beattie, and Bresnahan (BBB) scores and CatWalk gait analysis. Spinal cord (SC) histology was evaluated using hematoxylin and eosin and Nissl staining. NeuN-positive cells were quantified via immunofluorescence. Tumor necrosis factor-*α* (TNF-α), interleukin-6 (IL-6), and aquaporin-4 levels were determined through enzyme-linked immunosorbent assay. RhoA, ROCK2, Bax, Bcl-2, and cPLA2 mRNA levels were analyzed using real-time quantitative polymerase chain reaction. RhoA, ROCK2, Bax, Bcl-2, cPLA2, and p-cPLA2 protein expressions were analyzed using western blotting to investigate the impact of Tuina therapy on nerve regeneration and apoptosis regulation.

**Results:**

The TUI cohort showed better BBB scores and CatWalk results than the SNI cohort (all *p* < 0.001). Histological analysis revealed diminished inflammatory cell infiltration and increased neuronal survival. NeuN immunofluorescence indicated decreased motor neuron apoptosis in the anterior horn of the SC. Tuina therapy reversed TNF-*α*, IL-6, and aquaporin-4 levels (*p* < 0.01). The TUI cohort had lower mRNA expression of Bax, cPLA2, and ROCK2 (all *p <* 0.001), mRNA expression of RhoA (*p <* 0.01), and Bax, cPLA2, p-cPLA2, and RhoA/ROCK2 levels (all *p <* 0.001) than the SNI cohort. Conversely, mRNA and protein expression levels of Bcl2 were higher in the TUI cohort than in the SNI cohort (all *p <* 0.001).

**Conclusion:**

Tuina therapy improved motor function in SNI rats by inhibiting motor neuron apoptosis via cPLA2 regulation, potentially via the RhoA/ROCK2 signaling pathway.

## Introduction

Peripheral nerve injury (PNI) represents a prevalent traumatic disorder characterized by motor, sensory, and autonomic dysfunction (1, 2). Motor neuron apoptosis in the spinal cord (SC) is a major cause of motor impairment (3, 4). The sciatic nerve, a regenerative mixed peripheral nerve, transmits sensory signals through the dorsal root ganglion (DRG) and motor signals via axons in the ventral horn of the SC (5). Despite the regenerative potential of peripheral nerves, achieving a full recovery remains challenging (6, 7). If left untreated, PNI can lead to symptoms such as numbness, tingling, burning, and severe pain, often resulting in long-term disability and functional loss (8). In the United States and Europe, over 200,000 PNI-related surgeries are performed annually, incurring a total cost exceeding $100 billion (9), underscoring the need for innovative therapeutic strategies.

Tuina, a traditional Chinese therapeutic modality, is an effective, low-side-effect, complementary, and alternative therapy for PNI. It alleviates pain by releasing endogenous analgesic substances, promoting axonal and myelin regeneration through neurotrophic factor secretion, and suppressing excessive inflammation to treat nerve-related conditions (10, 11). Tuina relieves pain associated with SNI by modulating changes in 10 specific neurotransmitters in the SC (12). Additionally, owing to its ability to reduce neuronal apoptosis, Tuina is widely used for its neuroprotective effects against neurodegenerative diseases (13–15). This investigation examined the impact of Tuina therapy on SC inflammation and motor neuron apoptosis to improve motor dysfunction caused by sciatic nerve injury.

The local inflammatory response triggered by sciatic nerve injury is a key factor in spinal motor neuron apoptosis (16) because the release of inflammatory mediators creates a sustained inflammatory environment that affects the central nervous system and promotes motor neuron death (17). Cytosolic phospholipase A2 (cPLA2) is a principal mediator in inflammatory processes (18), and its phosphorylation intensifies this response. Conversely, suppressing the Ras homolog family member A/Rho-associated coiled-coil containing protein kinase 2 (RhoA/ROCK2) pathway reduces the release of pro-inflammatory factors and prevents apoptosis through mechanisms such as mitochondrial dysfunction and Bax/Bcl-2 signaling (19, 20). Therefore, targeting the RhoA/ROCK2 pathway is a promising strategy to reduce motor neuron apoptosis in the SC.

This study builds upon our prior RNA-seq evidence regarding Tuina therapy. Established research confirms that Tuina alleviates SNI-induced motor deficits by modulating apoptotic pathways and Ras signaling cascades (21, 22). Given that the Ras homolog family member A/Rho-associated coiled-coil containing protein kinase 2 (RhoA/ROCK2) pathway the RhoA/ROCK2 pathway functions as a key apoptosis regulator within the Ras superfamily (23), this study aims to assess changes in RhoA/ROCK2 pathway proteins and apoptotic effectors following Tuina intervention, to elucidate the mechanism by which Tuina ameliorates motor dysfunction after PNI.

The SNI model simulated clinical PNI, enabling evaluation of neuropathic changes and nerve regeneration. The “Three-Manipulation and Three-Acupoint” is a combination of manipulations and acupoints that we have studied and proven to be effective (1, 16, 22). Yinmen (BL37) at the sciatic nerve trunk projection (biceps femoris); Chengshan (BL57) at the tibial nerve projection (gastrocnemius); and Yanglingquan (GB34) at the common peroneal nerve projection (tibialis anterior). Following 20 intervention sessions, significant improvements in neuromuscular structure and demonstrated significant effects of the “Three-Manipulation and Three-Acupoint.”

Although the mechanical effects of Tuina on sciatic nerve injury are known to improve spinal motor neuron survival by inhibiting inflammation and apoptosis, whether this process is regulated by the cPLA2 and RhoA/ROCK2 signaling pathways remains unclear. To address these questions, we established SNI models in rats to assess the effects of Tuina on motor neuron protection, inflammation, apoptosis, and the cPLA2 and RhoA/ROCK2 pathways. This study is the first-time to investigate the involvement of the RhoA/ROCK2 signaling pathway in Tuina’s therapeutic effects.

## Methods

### Animals and groups

Male Sprague–Dawley rats aged 8 weeks (200 ± 10 g) were acquired from Beijing SPF Biotechnology Co., Ltd. (SCXK [Beijing] 2019–0010, Beijing, China). They were maintained within the barrier facility at the Beijing University of Chinese Medicine Animal Center under a 12-h light/dark cycle with unrestricted access to food and water, allowing a 7-day adaptation period before experimentation. In total, 36 rats were arbitrarily split into four experimental cohorts [blank control (CON), sham model (SHA), SNI, and TUI], with nine animals allocated to each cohort. All research protocols received authorization from the Animal Use and Management Committee of Beijing University of Chinese Medicine (approval number: BUCM-2023061906-2220), adhering to the 3Rs principles (Replacement, Reduction, Refinement) and in accordance with NIH protocols for laboratory animal maintenance and handling.

### Development of sciatic nerve compression model

**Figure 1:**
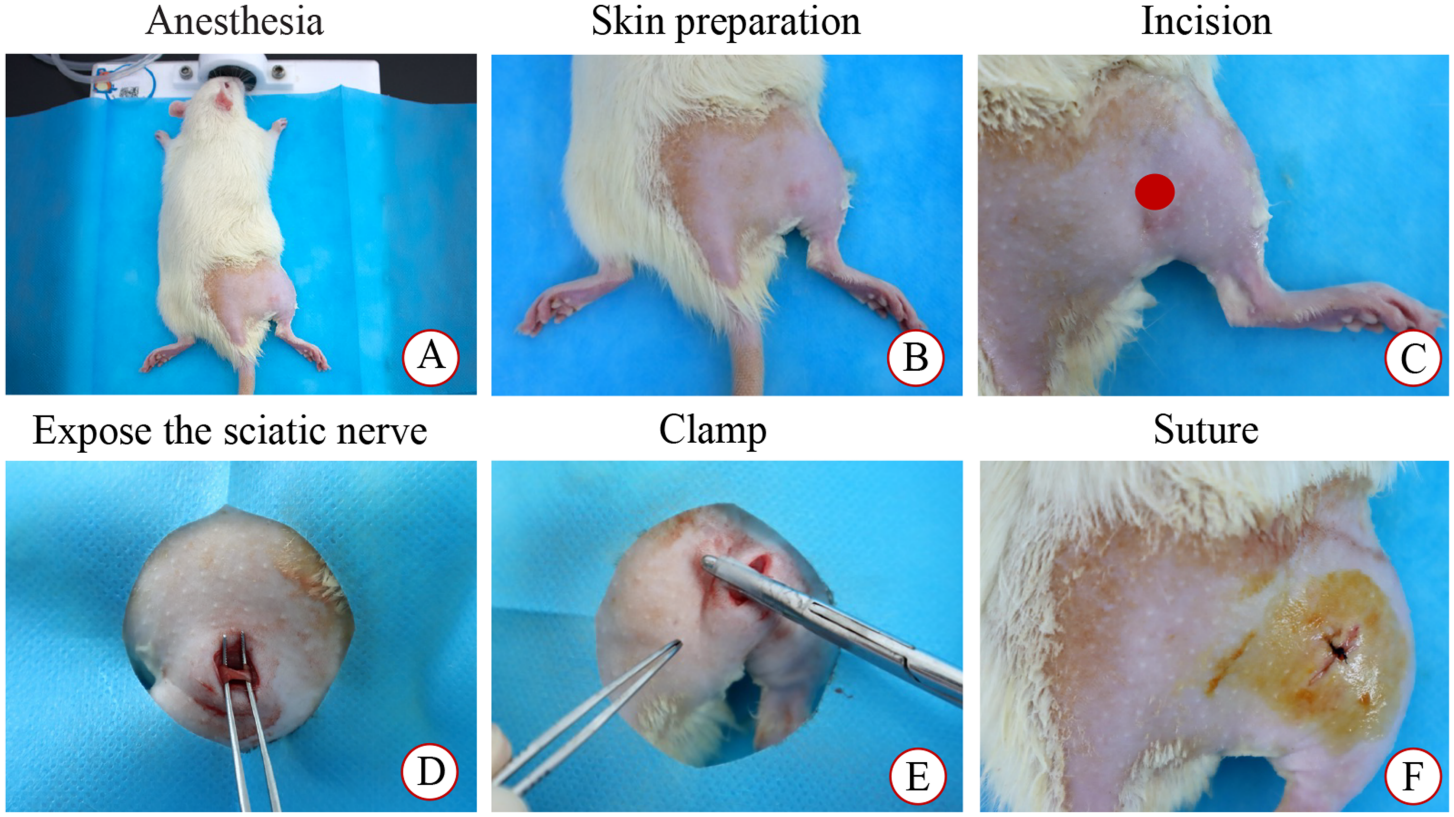
Modeling process. **(A)** Anesthesia; **(B)** Skin preparation and disinfection; **(C)** Incision; **(D)** Expose the sciatic nerve; **(E)** Clamp; **(F)** Suture.

The SNI rat model was developed as previously described (24). After a 7-day acclimation period, the right hind limbs of rats in the SHA, SNI, and TUI cohorts were shaved and under isoflurane anesthesia (R510-22-10, Shenzhen Ruowei Life Science Technology Co., Ltd., China). A 1 cm incision was made along the femur axis in the right sciatic nerve projection area to visualize the sciatic nerve. In the SHA cohort, the nerve was separated from the muscle without clamping, while in the SNI and TUI cohorts, a custom-made hemostatic clamp was applied to the sciatic nerve for 5 s with a pressure of 4 N. After modeling, the animals were arbitrarily assigned to the SNI or TUI cohort (Figure 1).

### Tuina therapy

**Figure 2:**
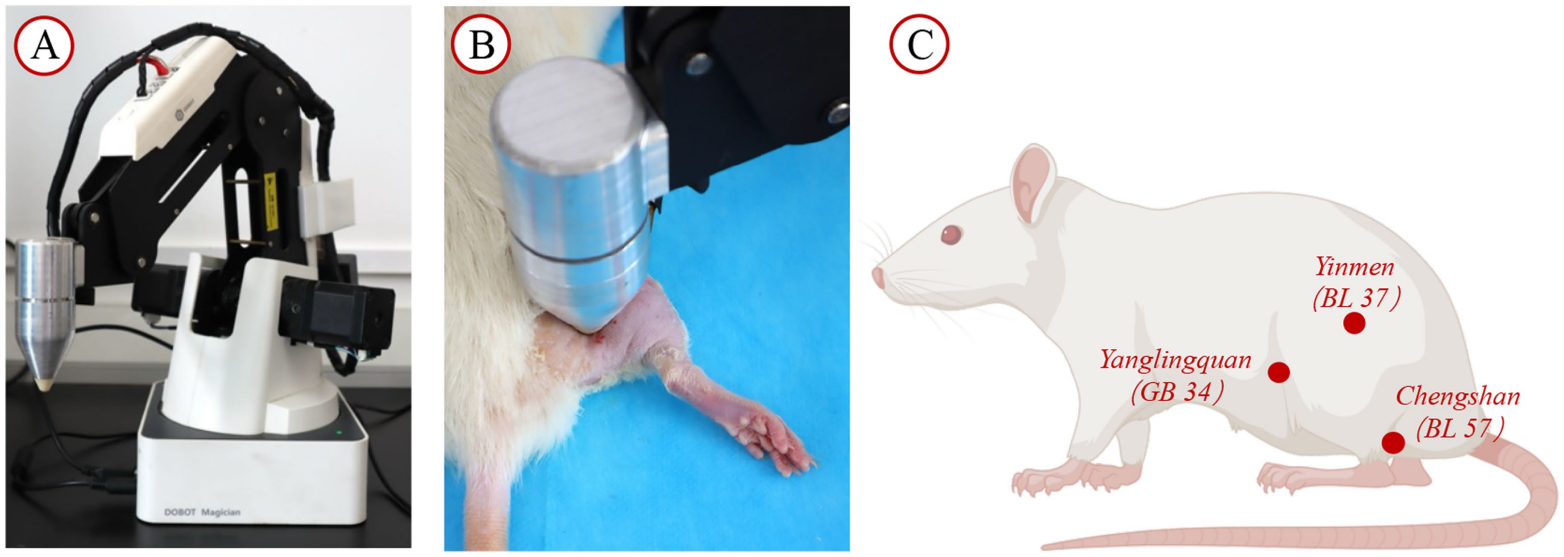
Intervention facilities and site diagram. **(A)** Tuina operation simulator; **(B)** SD rat placed on the rat platform for intervention with the instrument; **(C)** Diagram illustrating the acupoint locations.

The intervention commenced on the 7th day following model establishment. To ensure intervention quality control, all manipulations were performed exclusively by the same trained practitioner using the Intelligent Tuina device (Chinese Patent No. ZL202320511277.5). This device precisely regulated force, duration, and frequency parameters during “Three-Manipulation and Three-Acupoint” interventions, guaranteeing methodological consistency (25). The rats in the TUI cohort received Tuina therapy simulated using the Intelligent Tuina device (Chinese Patent No. ZL202320511277.5), employing the “Three-Manipulation and Three-Acupoint” technique (1, 26), which included pressing, plucking, and kneading on the acupoints Yinmen (BL37), Chengshan (BL57), and Yanglingquan (GB34). The pressure was set to 4 N, with a frequency of 90 cycles per minute (Figure 2). Each technique was applied for 1 min per acupoint, for a total of 9 min per session (1 min per technique × 3 techniques × 3 acupoints). The intervention was performed once daily, with a cycle of 10 sessions, followed by 1 day of rest, for a total treatment duration of 21 days. The control cohort underwent no intervention. The sham surgery and model cohorts underwent restraining interventions, with one session per day, each lasting 9 min, for 10 days, followed by 1 day of rest, for a total of 21 days.

### BBB locomotor scores

Motor function recovery in rats following SNI was assessed using the Basso, Beattie, and Bresnahan (BBB) scales. BBB assessments were conducted in an unrestricted environment before surgery, 1 day before the intervention, and on the 10th and 20th days post-intervention. Trained observers who were blinded to group allocations performed all evaluations. The scale ranges from 0 (complete paralysis) to 21 (normal movement), with scores between 1 and 20, providing a detailed classification based on rat activity. The evaluation considered factors such as limb joint movement, forelimb and hindlimb coordination, trunk stability, tail and body movements, and weight-bearing ability.

### Gait analysis

Gait analysis following intervention was conducted by investigators blinded to group assignments using the DigiGait™ Imaging System (MSI-DIG-AMW, Mouse Specifics, United States) to assess functional recovery in the affected limb after SNI. One week before surgery, the rats were acclimated to the equipment, with the treadmill gradually increasing in speed to 10 cm/s. The system automatically recorded the animal’s full movement and calculated the maximum contact area at the peak contact pressure of the right hind limb (paw area), the ratio of swing to stance phase duration (stance/swing), and the ratio of maximum stride area to stride duration (dA/dT).

### Sample collection and preparation

After completing the CatWalk gait analysis, the rats received anesthesia through an intraperitoneal administration of 1% pentobarbital sodium (57–33-0, Beijing Ouhuo Technology Co., Ltd., China), and the SC was subsequently dissected. Under a cold environment, the L4-6 segment of the SC was swiftly extracted. One portion of the SC was flash-frozen in liquid nitrogen and maintained at −80°C, while another portion was preserved in 4% paraformaldehyde for 24 h.

### Histochemical analysis

Histochemical evaluation was performed employing Nissl staining to examine variations in the SC tissue cavity regions, inflammatory cell infiltration, and neuronal apoptosis among the distinct cohorts. SC specimens were stabilized in 4% paraformaldehyde, subsequently washed, subjected to dehydration via an ethanol gradient, rendered transparent with xylene, and incorporated into paraffin. Paraffin blocks were sectioned to 4–5 μm thickness utilizing a Leica RM2235 microtome (Leica, Germany). Following deparaffinization, the sections underwent hematoxylin and eosin (H&E) and Nissl staining, proceeded by dehydration, transparency enhancement, and mounting with neutral resin. SC tissue morphology was assessed by investigators blinded to group assignments under an optical microscope (BX43, Olympus, Japan) at 200 × and 400 × magnifications, and 4–5 fields from each section were randomly selected for imaging. H&E was utilized to examine the overall structural alterations in the SC, and Nissl staining was used to evaluate neuronal survival.

### Immunofluorescence

After deparaffinizing and cleaning, the SC tissue sections were air-dried and blocked with 5% goat serum. The sections were subsequently maintained at 4°C overnight with a mouse anti-NeuN antibody (1:200 dilution, 66,836-1-1 g, Proteintech Group, Inc., China). The following day, the sections were washed five times with PBS for 8 min each. Subsequently, the sections were incubated for 60 min at room temperature with goat anti-mouse IgG H&L (1:500 dilution, AB0142, Shanghai Bowan Biotechnology Co., Ltd., Shanghai, China) in the dark. Cell nuclei underwent DAPI staining treatment for 10 min (ZKWB-0, Beijing Zhongke Wanbang Biotechnology Co., Ltd., Beijing, China), and the slides were sealed with an aqueous mounting medium. Images were analyzed by investigators blinded to group assignments utilizing a fluorescence microscope (TH4-200, Olympus, Japan) and ImageJ software (National Institutes of Health, United States).

### Enzyme-linked immunosorbent assay

Per the supplier’s protocols, ELISA kits for rat TNF-*α* (RGB-60080R, RegBio, China), IL-6 (RGB-60023R, RegBio, China), and aquaporin-4 (AQP-4) (RGB-60752R, RegBio, China) were used for detection. After anesthesia, an appropriate amount of the SC anterior horn tissue was collected, weighed, and homogenized with PBS at a 1:9 volume ratio employing a homogenizer (LANYI-GTM, Shanghai Lanyi, China). The mixture underwent centrifugation at 3,000 rpm for 20 min, and the supernatant was extracted for analysis. The assay protocol encompassed standard dilution, sample addition, washing, color development, and reaction termination. The absorbance was determined at 450 nm utilizing a microplate reader (Thermo Multiskan MK3, Thermo Fisher Scientific, United States). A standard curve was developed, and TNF-*α*, IL-6, and AQP-4 protein levels in the samples were ascertained.

### Real-time quantitative polymerase chain reaction

SC tissue RNA was procured through TRIzol extraction methodology, and the RNA quality and concentration were evaluated utilizing a nucleic acid quantification device (Unano-1000, UMI Instruments, China). Complementary DNA was prepared employing a reverse transcription system (A502, EXONGEN, Israel), before quantitative fluorescence analysis. In summary, RNA isolation was executed per the supplier’s protocols to avoid RNAse contamination. The RNA purity and yield were assessed employing a nucleic acid concentration measuring instrument. The isolated RNA underwent reverse transcription to generate cDNA utilizing a reverse transcription system. Quantitative PCR analysis was executed on a CFX Connect™ RT-PCR instrument (1,855,200, Bio-Rad, United States), with cDNA as the amplification template. The thermal cycling parameters encompassed initial denaturation at 95°C for 5 min, succeeded by 40 cycles of denaturation (95°C, 10 s), annealing (58°C, 20 s), and extension (72°C, 20 s). A melt-curve analysis was subsequently executed. The transcript levels of RhoA, ROCK2, Bax, Bcl-2, and cPLA2 were determined through the 2^-△△Ct^ methodology, utilizing glyceraldehyde-3-phosphate dehydrogenase (GAPDH) as the internal control for standardization. The primer sequences utilized in these experiments are depicted in Appendix 1.

### Western blotting

For western blot analysis, 100 mg of SC tissue was subjected to homogenization utilizing immunoprecipitation lysis buffer (P0013; Shanghai Biyuntian Biotechnology Co., Ltd., Shanghai, China) to obtain total protein. Protein concentration was quantified using the BCA assay for subsequent comparisons. Briefly, 20 μg of total protein was separated by SDS-PAGE, and the resulting gel was transferred onto a PVDF membrane following methanol immersion for 1 min. The transfers were conducted at 100 V for 1 h. Subsequently, the membranes underwent blocking at room temperature for 30 min with a rapid blocking solution. The blocked membrane underwent overnight incubation at 4°C with the following primary antibodies: rabbit anti-GAPDH (1:3000, 10,494-1-AP, Proteintech Group, Inc., China), rabbit anti-RhoA (1:1000, 10,749-1-AP, Proteintech Group, Inc., China), rabbit anti-ROCK2 (1:1000, 21,645-1-AP, Proteintech Group, Inc., China), rabbit anti-Bax (1:1000, 60,267-1-Ig, Proteintech Group, Inc., China), rabbit anti-Bcl-2 (1:1000, 26,593-1-AP, Proteintech Group, Inc., China), rabbit anti-cPLA2 (1:1000, AF6329, Jiangsu Qinke Bio Research Center Co., Ltd., China), and rabbit anti-p-cPLA2 (1:1000, AF3329, Jiangsu Qinke Bio Research Center Co., Ltd., China). The following day, the membrane underwent a triple wash process using 1 × TBST, with each wash lasting 10 min, followed by a 60-min exposure to horseradish peroxidase-linked secondary antibody, goat anti-rabbit IgG H&L (1:3000, MD912565, Kangtai Medical Testing Service, Hebei, China). Following three additional TBST rinses, the membrane received treatment with enhanced chemiluminescence substrate, combined at equal proportions, and applied for 1 min. Image acquisition was performed utilizing a ChemiDoc MP imaging system (1,708,280, Bio-Rad, United States).

### Statistical analysis

**Figure 3:**
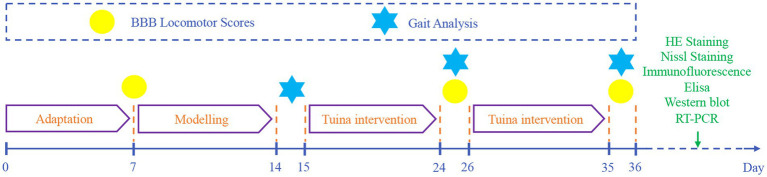
Flowchart of animal experiment.

Statistical analyses were performed using SPSS Statistics 26.0 (IBM Corp., Armonk, NY, United States). Continuous data are expressed as mean ± standard deviation. Normality was assessed using Shapiro–Wilk tests. For normally distributed data, intergroup differences were analyzed by one-way ANOVA followed by LSD *post hoc* tests when variance homogeneity held (verified by Levene’s test). Under variance heterogeneity, Tamhane’s T2 method was applied for pairwise comparisons. Non-normally distributed data were analyzed using Kruskal-Wallis tests with Dunn’s *post hoc* correction. Statistical significance was defined at *p* < 0.05 (Figure 3).

## Results

### TUI therapy enhanced the functional recovery of the sciatic nerve in SNI rats

No signs of redness or swelling were observed at the injury site following model induction, and the postoperative recovery was satisfactory. On the 7th day after SNI surgery, rats were unable to support their body weight on the injured limb, exhibited limping or dragging behavior, and experienced declined motor coordination and fine motor skills, confirming the success of model induction.

**Figure 4:**
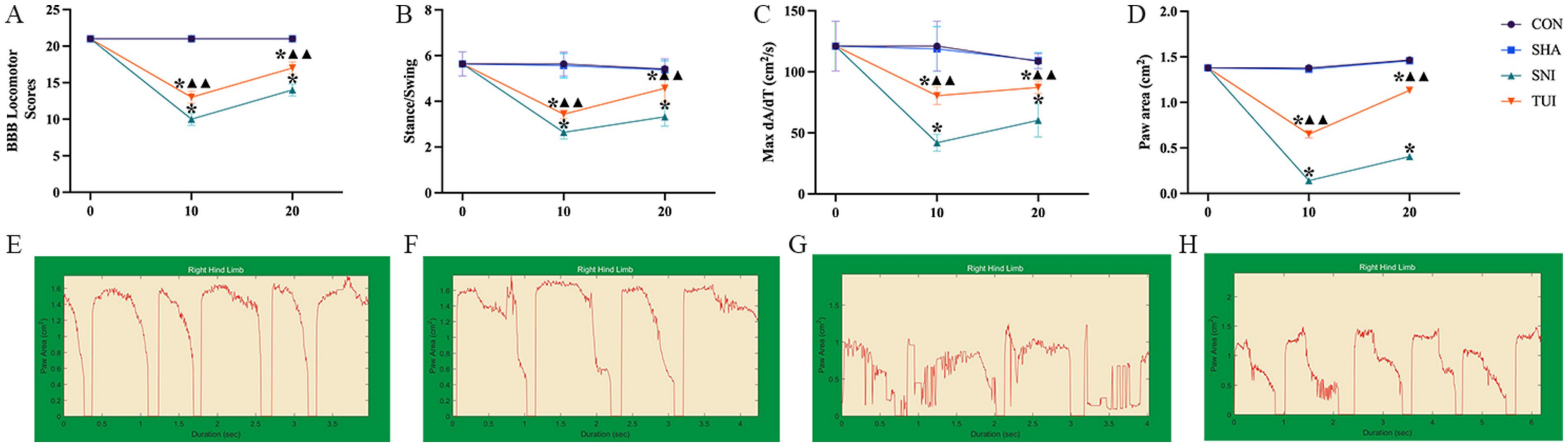
BBB scores and gait analysis. **(A)** BBB Locomotor Scores; **(B)** Stance/Swing Ratio; **(C)** Max dA/dT; **(D)** Paw Area. **(E–H)** Screen-captured images of DigiGait™ periodic waveforms representing stance and swing phases of stride of rat hind limb. **(E)** CON cohorts; **(F)** SHA cohorts; **(G)** SNI cohorts; **(H)** TUI cohorts. (*n* = 6) *p < 0.001 vs. SHA; ▲compared to SNI, ▲▲*p* < 0.001.

We assessed the recovery of neurological function and hind limb motor ability using the BBB scoring systems. No notable variations in BBB scores were detected between the CON and SHA cohorts after the 10th and 20th interventions. In contrast, the SNI cohort’s BBB scores were markedly lower than those of the CON and SHA cohorts (*p* < 0.001). After the 10th and 20th interventions, the TUI cohort showed significantly improved BBB scores compared with the SNI cohort (*p* < 0.001). However, the scores remained markedly diminished compared with those of the SHA cohort. These results indicate that Tuina therapy improves motor function following SNI (Figure 4A).

Stability and coordination of rat movements were evaluated by measuring paw area, stance/swing ratio, and maximum acceleration/time ratio. Compared with the CON and SHA cohorts, the SNI cohort exhibited markedly reduced paw area, stance/swing ratio, and maximum acceleration/time ratio (all *p* < 0.001). After the 10th and 20th interventions, the TUI cohort showed significant improvements in these parameters compared with the SNI cohort (*p* < 0.001). These observations suggest that Tuina intervention promotes hind limb stability and coordination during movement, enhancing the overall motor function in rats (Figures 4A–D). DigiGait™ periodic waveforms of rats right hind limb are presented in Figures 4E–H.

### Tuina therapy alleviated inflammatory response

**Figure 5:**
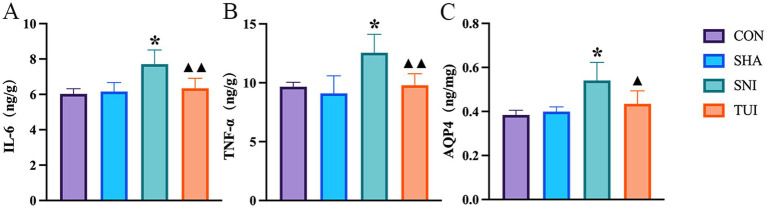
Levels of IL-6, TNF-*α*, and AQP-4 in spinal cord of rats after Tuina intervention. Levels of IL-6 **(A)**, TNF-α **(B)**, and AQP-4 **(C)** in spinal cord tissue, measured by enzyme-linked immunosorbent assay (ELISA). Data are presented as mean ± standard deviation. (*n* = 6) *p < 0.001 vs. SHA; ▲compared to SNI, ▲*p* < 0.01, ▲▲*p* < 0.001.

ELISA was applied to measure inflammatory factor levels in SC tissue to assess the inflammatory response. ELISA results showed heightened tumor necrosis factor-*α* (TNF-α), interleukin-6 (IL-6), and AQP-4 levels in the model cohort. Tuina treatment notably reduced these inflammatory cytokine levels in the SC (*p* < 0.01). These observations suggest that Tuina treatment alleviated the inflammation induced by sciatic nerve injury (Figures 5A–C).

### H&E staining and Nissl staining

**Figure 6:**
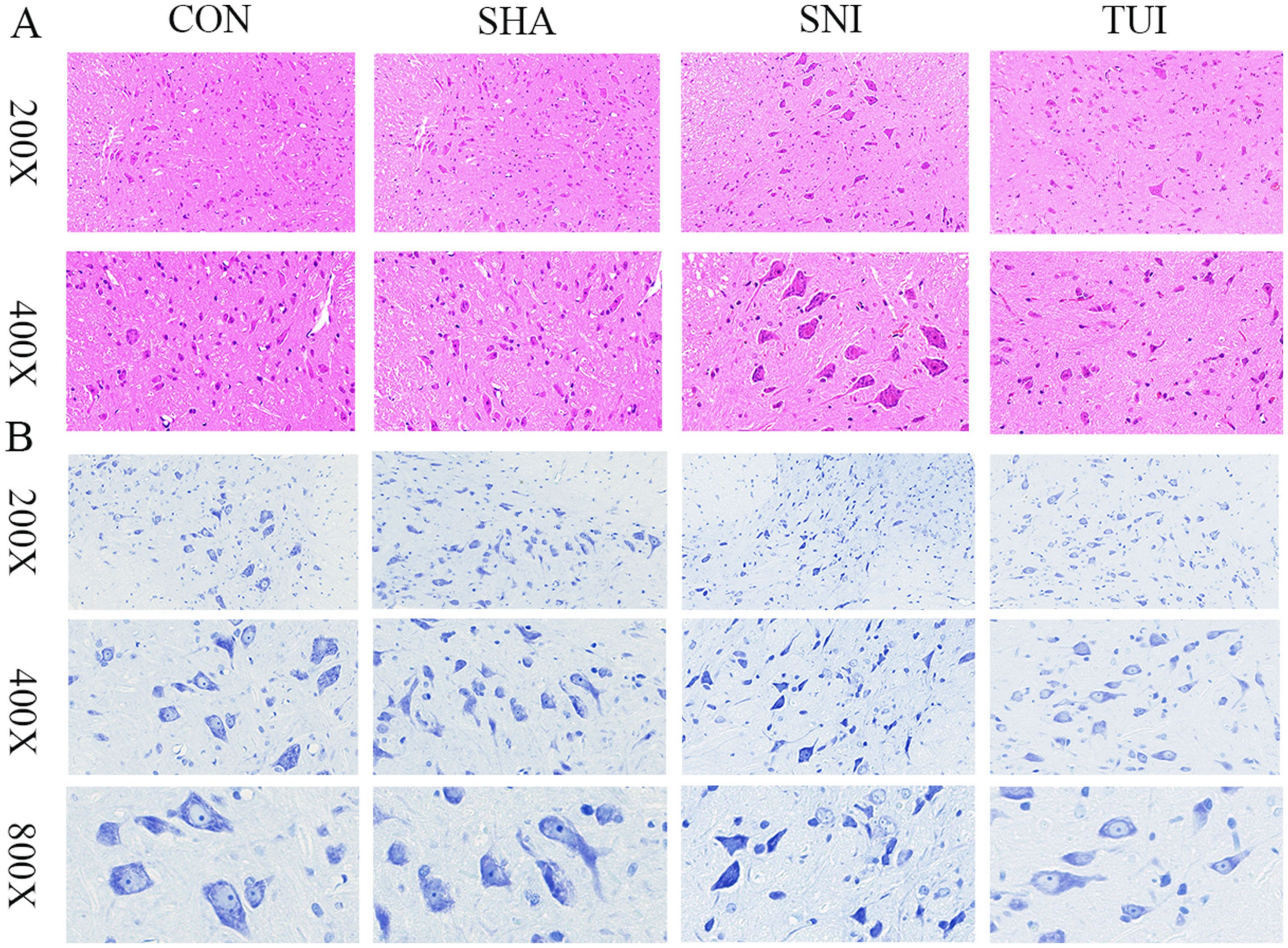
Spinal cord histopathology: Nissl and H&E staining. **(A)** Spinal cord tissue section; magnification: 200 × (scale bar = 50 μm), 400 × (scale bar = 20 μm). **(B)** Nissl staining of spinal cord tissue; magnification: 200 × (scale bar = 100 μm), 400 × (scale bar = 50 μm), 800 × (scale bar = 25 μm).

Histological analysis was executed using H&E staining to examine structural changes in the SC tissue at the injury site. The outcomes indicated that SC tissue in the CON and SHA cohorts appeared mostly normal, without significant pathological alterations. In contrast, rats in the SNI cohort exhibited severe damage to SC segments at the sciatic nerve injury site, including neuronal dissolution, vacuole formation, neuronal degeneration, and nuclear pyknosis, accompanied by significant inflammatory cell infiltration and focal hemorrhage. Furthermore, Tuina treatment reduced vacuole-like changes and alleviated inflammatory cell infiltration at the injury site in the SNI cohort. These observations suggest that Tuina therapy mitigated the structural changes in the SC caused by sciatic nerve injury (Figure 6A).

The survival status of motor neurons within the anterior horn of the SC was assessed through Nissl staining. The examination revealed that motor neurons displayed normal morphology in both CON and SHA cohorts, characterized by transparent cytoplasm and regularly shaped nuclei. In comparison, motor neuron counts were notably decreased in the SNI cohort, with certain neurons demonstrating vacuolation and condensed nuclei. The TUI cohort exhibited more distinct Nissl bodies and less pronounced nuclear condensation than the SNI cohort. These observations indicate that Tuina administration enhances neuronal viability and regeneration (Figure 6B).

### Tuina therapy improved cell apoptosis

**Figure 7:**
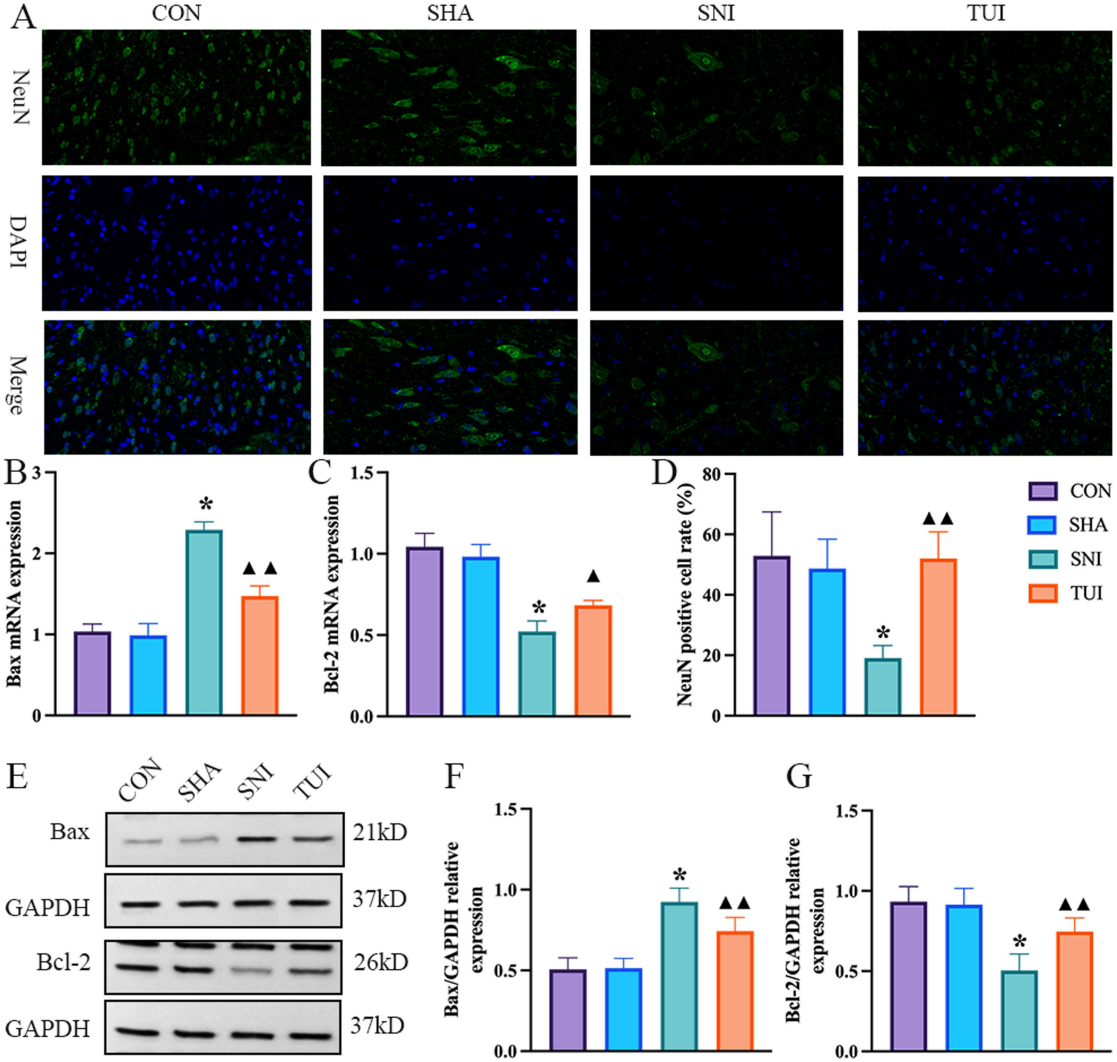
Immunofluorescence, mRNA expression levels, and apoptosis-related proteins. **(A)** Immunofluorescence staining of NeuN protein in spinal cord tissue. Magnification: 400 × (scale bar = 50 μm). **(B,C)** Real-time RT-PCR analysis of Bax **(B)** and Bcl-2 **(C)** mRNA expression levels. (*n* = 6) *p < 0.001 vs. SHA; ▲compared to SNI, ▲*p* < 0.01, ▲▲*p* < 0.001. **(D)** Fluorescence intensity of NeuN in spinal cord tissue (*n* = 3). **(E)** Quantification of specific signal intensity. **(F,G)** Relative protein expression of Bax **(F)** and Bcl-2 **(G)** in the spinal cord. Data are presented as mean ± standard deviation. (*n* = 6) *p < 0.001 vs. SHA; ▲compared to SNI, ▲*p* < 0.01, ▲▲*p* < 0.001.

Immunofluorescence staining was used to assess apoptosis in SC tissue. NeuN immunofluorescence staining showed that the nuclei were stained blue with DAPI, and NeuN was stained green to label the motor neurons in the anterior horn of the SC. NeuN expression was markedly lower in the SNI cohort than in the CON and SHA cohorts (*p* < 0.001). NeuN distribution was more pronounced in the TUI cohort, with markedly higher fluorescence intensity than that in the SNI cohort (*p* < 0.001). The findings demonstrate that Tuina treatment successfully improved motor neuron apoptosis following sciatic nerve injury (Figures 7A,D).

The expression levels of apoptosis-associated genes were analyzed through RT-PCR methodology. The SNI cohort exhibited a marked elevation in Bax mRNA levels compared with both CON and SHA cohorts (*p* < 0.001), while Bcl-2 expression demonstrated significant downregulation (*p* < 0.001). Conversely, the TUI cohort showed diminished Bax mRNA levels alongside enhanced Bcl-2 expression (*p* < 0.01, Figure). These findings indicated that Tuina suppressed apoptotic processes via modulation of apoptosis-related gene expression (Figures 7B,C).

Western blot analysis evaluated the impact of Tuina extract on proteins associated with apoptosis. Analysis revealed that Bax protein levels within SC tissues exhibited significant elevation in both SNI and TUI cohorts compared with those in the SHA cohort (*p* < 0.001). Nevertheless, Bax levels demonstrated a marked reduction in the TUI cohort compared with that in the SNI cohort (*p* < 0.001). Bcl-2 expression displayed decreased levels in the SNI cohort relative to the SHA cohort (*p* < 0.001) while showing enhanced expression in the TUI cohort compared with that in the SNI cohort (*p* < 0.001). The observed data indicates that Tuina administration diminished the Bax/Bcl-2 ratio, consequently mitigating apoptosis post-SNI (Figures 7E–G).

### Effect of Tuina therapy on the gene and Protein expression related to the cPLA2 and RhoA/ROCK2 pathways

**Figure 8:**
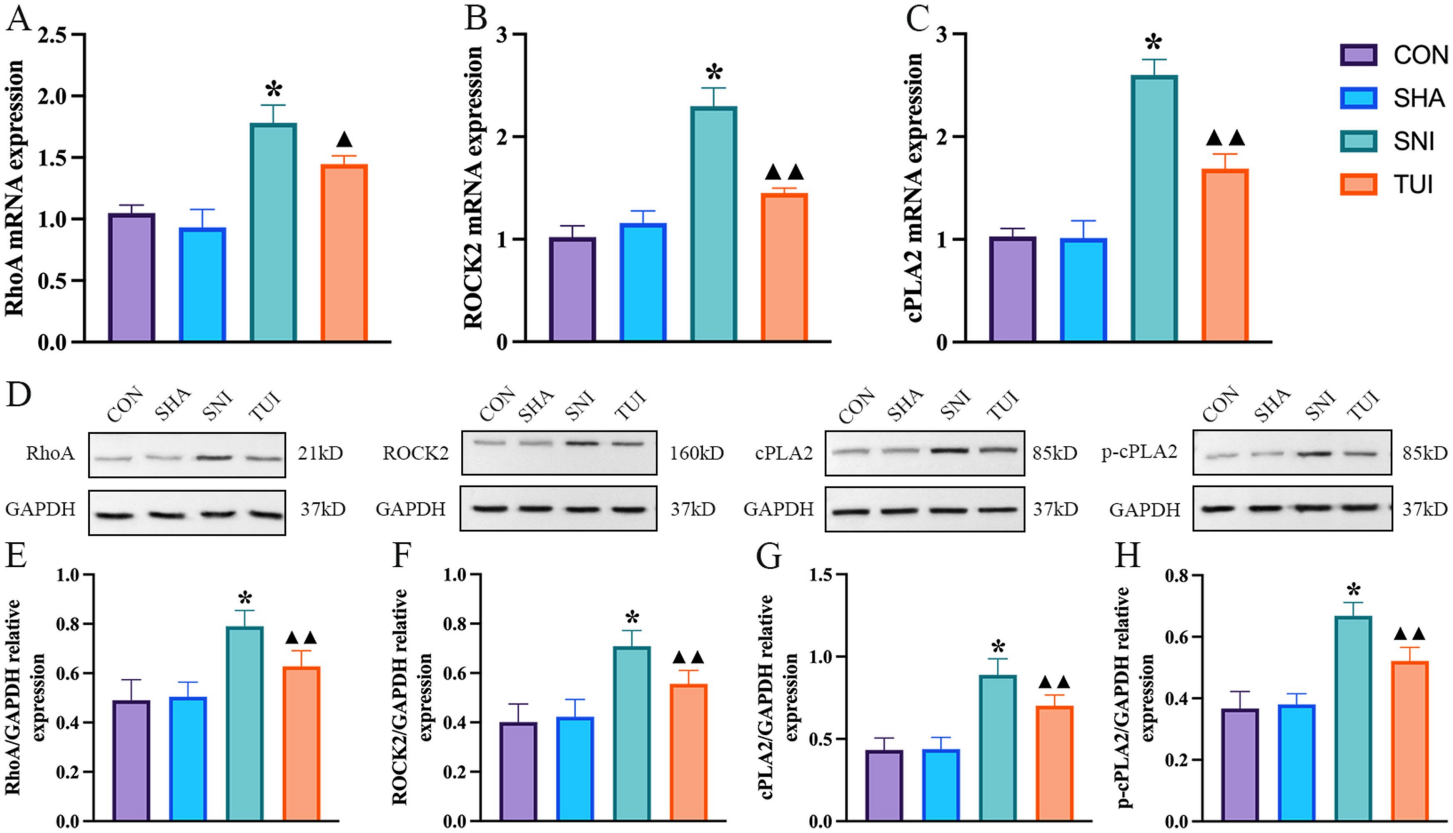
Tuina-regulated factors associated with cPLA2 and RhoA/ROCK2 pathways levels. **(A–C)** mRNA expression levels of RhoA **(A)**, ROCK2 **(B)**, and cPLA2 **(C)** detected by real-time RT-PCR. **(D)** Quantification of specific signal intensities. **(E–H)** Relative protein expression of RhoA **(E)**, ROCK2 **(F)**, cPLA2 **(G)**, and p-cPLA2 **(H)** in spinal cord tissue. Data are presented as mean ± standard deviation. (*n* = 6) *p < 0.001 vs. SHA; ▲compared to SNI, ▲*p* < 0.01, ▲▲*p* < 0.001.

RT-PCR examination of SC tissue indicated alterations in the RhoA/ROCK2 signaling cascade. Compared to the SNI cohort, the mRNA expression level of RhoA in the TUI cohort decreased (*p* < 0.01; Figure 8A), and the ROCK2 was significantly decreased (*p* < 0.001; Figure 8B), and the cPLA2 was significantly decreased (*p* < 0.001; Figure 8C).

Western blot analysis suggested alterations in the RhoA/ROCK2 signaling cascade (Figure 8D). Compared to the SNI cohort, the protein abundance of RhoA in the Tuina treatment cohort showed a significant decreased (*p* < 0.001; Figure 8E), and ROCK2 in the TUI showed a significant decreased (*p* < 0.001; Figure 8F), and cPLA2 in the TUI showed a significant decreased (*p* < 0.001; Figure 8G), and p-cPLA2 in the TUI showed a significant decreased (*p* < 0.001; Figure 8H).

## Discussion

Tuina, a non-pharmacological therapy widely used in clinical settings for PNI management, offers distinct advantages including rapid onset, absence of drug dependence, and procedural simplicity (27). Evidence confirms its efficacy in promoting post-PNI neurological recovery and improving spinal cord (SC) tissue morphology (28). As a form of traditional Chinese external therapy, Tuina achieves neural functional restoration through physical intervention, representing a unique therapeutic approach complementary to conventional treatments (29). The “Three-Manipulation and Three-Acupoint” intervention method, which combines proven techniques and acupoints, is effective through experimental validation (30). By applying the three techniques—pressing, picking, and kneading—on the Yinmen (BL 37), Chengshan (BL 57), and Yanglingquan (GB 34) acupoints, behavioral recovery was promoted effectively in SNI model rats. Moreover, after 20-day treatment, motor function improved in the TUI cohort, confirming the therapeutic efficacy of the “Three-Manipulation and Three-Acupoint” approach. This investigation evaluated the motor function recovery in SNI-induced rats through BBB scoring and CatWalk gait analysis. The findings demonstrated that the “Three-Manipulation and Three-Acupoint” Tuina intervention effectively alleviated motor dysfunction in the SNI rats, gradually restoring their function to normal levels.

### Local inflammation-induced cell apoptosis is crucial in SNI

Apoptosis, induced by local inflammatory responses, is pivotal in SNI. Following SNI, pro-inflammatory cytokines released at the injury site activate neuroinflammatory pathways, exacerbating nerve damage and potentially leading to motor neuron apoptosis in the anterior horn of the SC, thereby impairing motor function (31, 32). Excessive immune responses in the anterior horn of the SC trigger cellular stress, promote neuronal apoptosis, and worsen motor function loss (33). Therefore, reducing the inflammatory response is a key strategy for protecting motor neurons and promoting nerve regeneration and repair. Inhibiting inflammation not only reduces neuronal apoptosis but also facilitates the recovery of motor function (34). In summary, excessive inflammation following SNI disrupts the homeostasis of motor neurons in the anterior horn of the SC, amplifying neuronal apoptosis and motor dysfunction. Modulating the spinal inflammatory microenvironment and cell apoptosis is promising for improving motor impairments resulting from SNI. Tuina therapy effectively reduced motor neuron apoptosis after SNI, as evidenced by increased NeuN expression, decreased Bax levels, and elevated Bcl-2 mRNA and protein levels, modulating the Bax/Bcl-2 ratio to protect motor neurons and improve survival.

### The cPLA2 pathway links inflammation and cell apoptosis

cPLA2 is crucial in regulating neuroinflammatory responses (35). TNF-*α* and IL-6 are key inflammatory markers in the nerve injury response. These cytokines activate the cPLA2 signaling pathway, enhancing its enzyme activity and promoting the synthesis of inflammatory mediators, encompassing prostaglandins and leukotrienes, further exacerbating local nerve inflammation (36). cPLA2 phosphorylation promotes the synthesis of these mediators and may also influence the expression of AQP4 (37). Abnormal AQP4 expression increases water transport, leading to SC edema. Edema worsens nerve tissue damage and potentially disrupts the blood–brain barrier, allowing more inflammatory cells to infiltrate and cause further neuronal injury. The investigation measured TNF-α, IL-6, and AQP4 expression levels in SC tissue, finding markedly elevated levels in the SNI cohort. In contrast, after 20 sessions of Tuina intervention, cPLA2 protein and mRNA expression decreased. Additionally, after 20 interventions, the SC tissue structure was relatively intact, and the infiltration of inflammatory cells was alleviated, suggesting that Tuina therapy reduces inflammation, inhibits the excessive expression of AQP4, and mitigates edema and nerve damage.

### Role of the RhoA/ROCK2 signaling pathway

The RhoA/ROCK2 pathway is a critical signaling pathway in neuronal apoptosis in neurological disorders (38, 39). cPLA₂, which mediates inflammatory and apoptotic processes, functions as a key effector in this pathway (40). Inhibition of RhoA/ROCK2 signaling reduces expression of downstream mediators, including pro-inflammatory cytokines (TNF-*α*, IL-6), the water channel protein AQP4, and the apoptosis regulator Bcl-2. RT-PCR and western blot analyses revealed that Tuina reduced Bax levels and increased Bcl-2 levels, diminishing the Bax/Bcl-2 ratio. Additionally, Tuina suppressed the mRNA and protein levels of cPLA2 and RhoA/ROCK2, suggesting that Tuina alleviates apoptosis and protects motor neurons by modulating the RhoA/ROCK2 signaling pathway.

Consistent with previous studies, the “Three-Manipulation and Three-Acupoint” Tuina intervention potentially suppresses the production of inflammatory mediators TNF-α and IL-6, reduce cPLA2 activity, and regulate the levels of AQP4. This intervention appeared to alleviate excessive activation of the RhoA/ROCK2 signaling pathway, decrease the Bax/Bcl-2 ratio, reduce apoptosis of motor neurons in the SC anterior horn, and improve the local inflammatory microenvironment in the SC. Although these outcomes suggest that Tuina intervention accelerates functional recovery following nerve injury, its clinical potential in nerve repair remains unclear.

## Conclusion

The “Three-Manipulation and Three-Acupoint” Tuina intervention could effectively promote motor function recovery after SNI, as demonstrated by improvements in BBB scores and CatWalk analysis. Tuina therapy could modulate the release of cPLA2 and inhibit the RhoA/ROCK2 signaling pathway, thereby reducing inflammation and motor neuron apoptosis in the SC to functional repair.

This research investigated the effects of Tuina regarding motor neuron apoptosis in the SC following SNI, emphasizing its therapeutic potential in nerve repair. Future research will explore whether SC tissue recovery can promote nerve repair and further elucidate the repair mechanisms of Tuina therapy. Although the present work examined Tuina’s impact on motor neuron apoptosis post-SNI, nerve damage constitutes the fundamental etiology. Thus, future investigation will determine whether recovery from such apoptosis facilitates neural repair or influences its efficacy, either beneficially or adversely. The results establish substantial scientific support for implementing Tuina in clinical treatments of PNI.

## Data Availability

The datasets presented in this study can be found in online repositories. The names of the repository/repositories and accession number(s) can be found in the article/Supplementary material.
