# Supplementary figures and images for: Chinese massage therapy (Tuina) inhibits motor neuron apoptosis in rats with sciatic nerve injury by regulating the cPLA2 and RhoA/ROCK2 signaling pathways
Source: Front Neurol. 2025 Jul 30;16:1622602. doi: 10.3389/fneur.2025.1622602 (PMC12344559; doi:10.3389/fneur.2025.1622602)

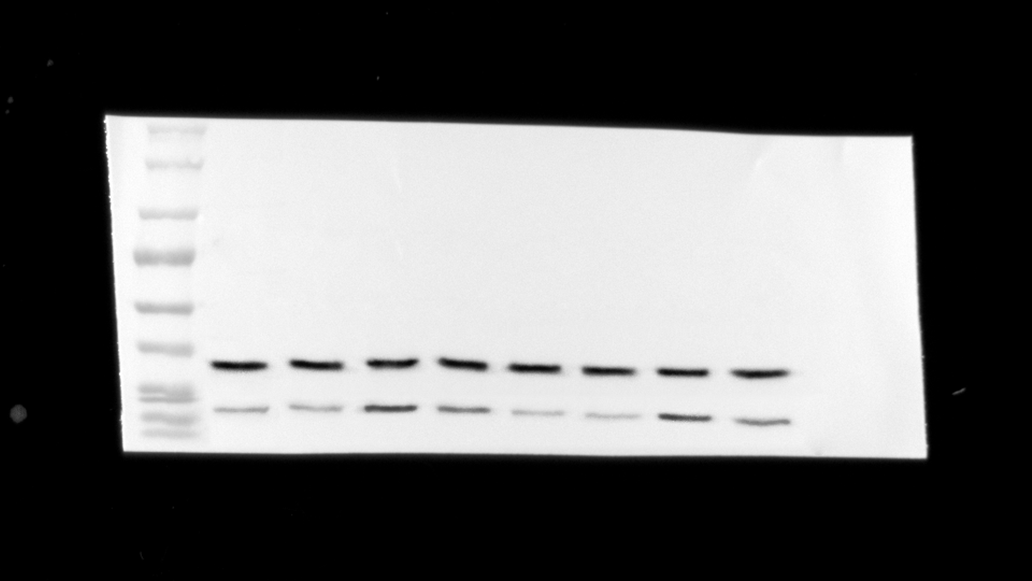

Supplement: Supplementary file 1 [file Image_1.TIF]

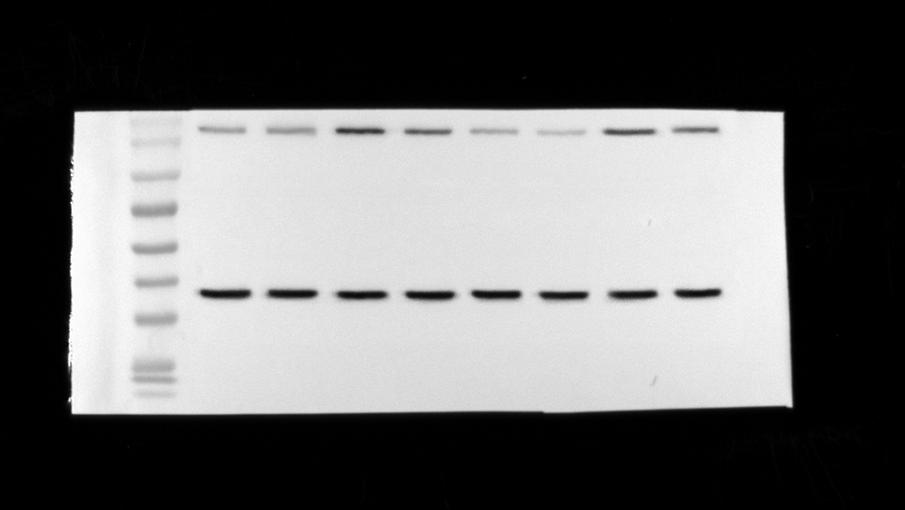

Supplement: Supplementary file 2 [file Image_2.TIF]

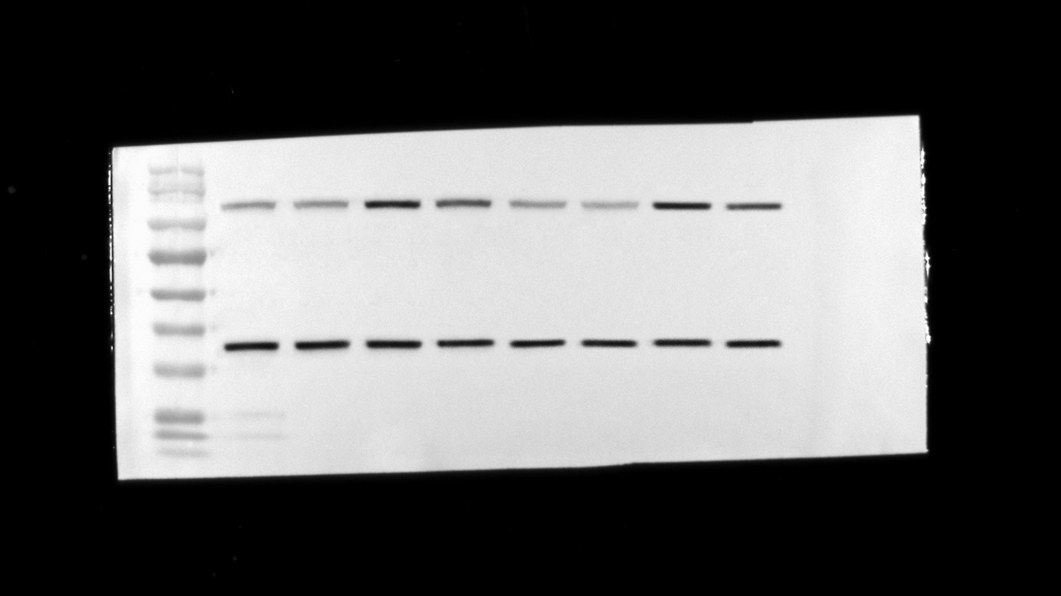

Supplement: Supplementary file 3 [file Image_3.TIF]

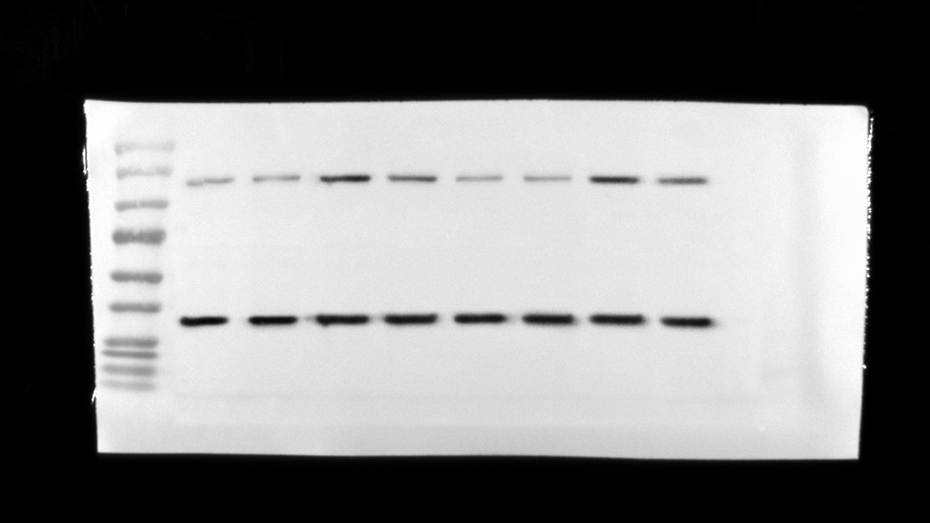

Supplement: Supplementary file 4 [file Image_4.TIF]

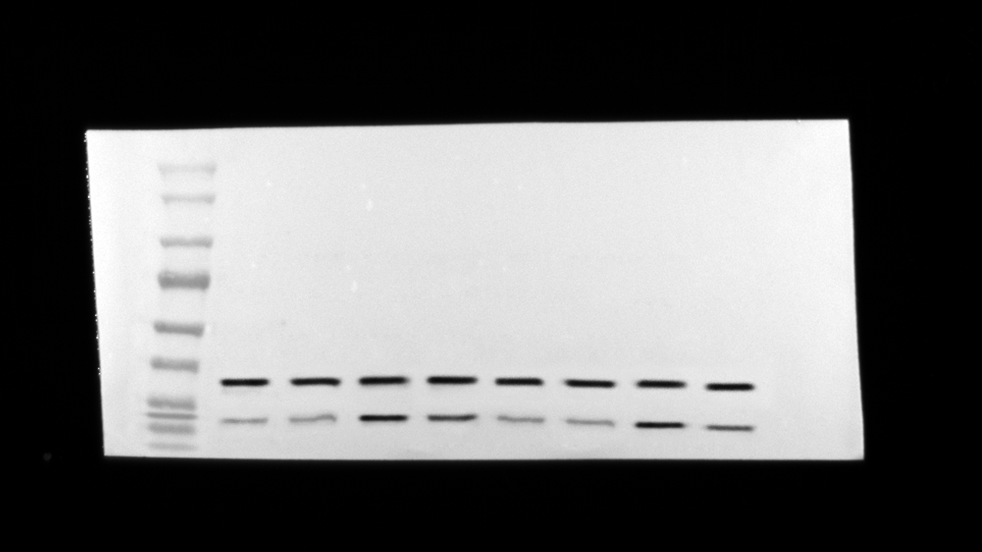

Supplement: Supplementary file 5 [file Image_5.TIF]

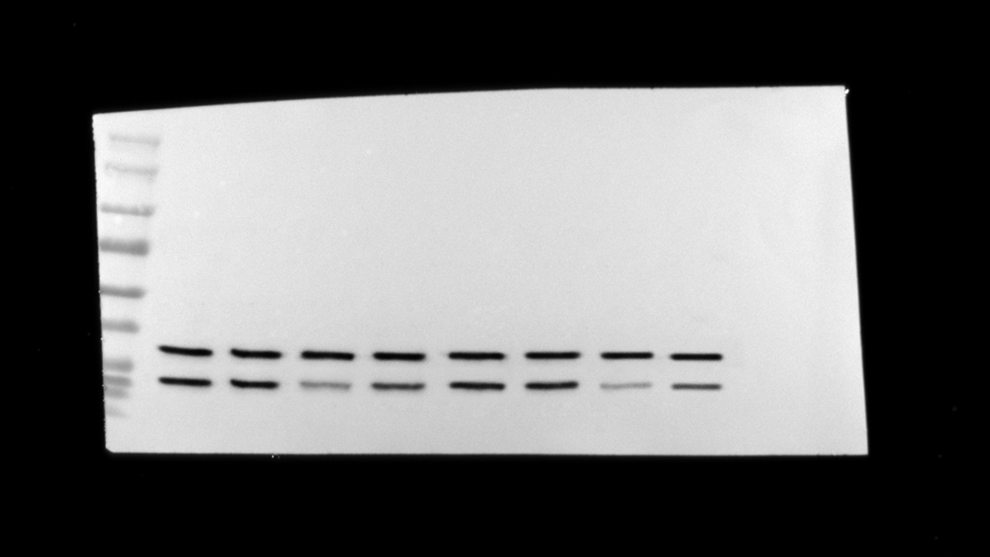

Supplement: Supplementary file 6 [file Image_6.TIF]
